# FN1 promotes prognosis and radioresistance in head and neck squamous cell carcinoma: From radioresistant HNSCC cell line to integrated bioinformatics methods

**DOI:** 10.3389/fgene.2022.1017762

**Published:** 2022-09-21

**Authors:** Xiaojun Tang, Qinglai Tang, Xinming Yang, Zi-An Xiao, Gangcai Zhu, Tao Yang, Qian Yang, Ying Zhang, Shisheng Li

**Affiliations:** Department of Otolaryngology Head and Neck Surgery, The Second Xiangya Hospital of Central South University, Changsha, China

**Keywords:** head and neck squamous cell carcinoma, radioresistance, bioinformatics methods, radioresistant HNSCC cell line, prognosis

## Abstract

**Background:** Radioresistance in head and neck squamous cell carcinoma (HNSCC) patients means response failure to current treatment. In order to screen radioresistant biomarkers and mechanisms associated with HNSCC, differentially expressed genes (DEGs) associated with radioresistance in HNSCC were investigated.

**Methods:** The HNSCC cell line with radioresistance, Hep2-R, was established and detected the radiosensitivity using MTT, colony formation assay and flow cytometry analysis. Clariom™ D chip was applied to compare DEGs between Hep2 and Hep2-R groups and build the differential gene expression profiles associated with radioresistance in HNSCC. Bioinformatic analysis were used to find biological functions and pathways that related to radioresistance in HNSCC, including cell adhesion, cytochrome P450 and drug metabolism. Gene Expression Omnibus (GEO) datasets were selected to verify DEGs between HNSCC radioresistant cells and tissues. The representation of DEGs were validated between HNSCC patients with complete response and post-operative radiation therapy failure. In addition, we evaluated the clinical prognosis of DEGs using The Cancer Genome Atlas (TCGA) database.

**Results:** 2,360 DEGs (|Fold Change|>1.5, *p* < 0.05) were identified between Hep2 and Hep2-R, including 1,144 upregulated DEGs and 1,216 downregulated DEGs. They were further verified by HNSCC radioresistant cells and tissues in GEO. 13 radioresistant DEGs showed same difference in expression level between cells and tissues. By comparing 13 DEGs with HNSCC patients, upregulations of FN1, SOX4 and ETV5 were found identical with above results. Only FN1 was a prognostic indicator of HNSCC in TCGA.

**Conclusion:** FN1 is the potential novel biomarker for predicting poor prognosis and radioresistance in HNSCC patients. Overexpression of FN1 plays an important role in the tumorigenesis, prognosis and radioresistance of HNSCC.

## Introduction

Head and neck squamous cell carcinoma (HNSCC) is seventh malignancies globally, which develops from mucosal epithelium in the nasal cavity, oral cavity, pharynx and larynx (Castellanos and Pan, 2016; Johnson et al., 2020; Pei et al., 2020). Over 60% patients with HNSCC are diagnosed at an advanced stage resulting in poor prognosis. Treatments including surgery, radiotherapy, chemotherapy and targeted therapy can reduce recurrence and prolong survival, while resistance to radiotherapy restricts the 5-year survival rate of HNSCC patients (Porcheri and Mitsiadis, 2021).

For HNSCC patients who are not suitable for surgery (advanced stage or co-existing diseases that cannot tolerate surgery), radiotherapy is a more suitable treatment (Jones et al., 2016; Kerawala et al., 2016; Mehanna et al., 2016; Pracy et al., 2016). Tumor tissues are more sensitive to radiation comparing with normal tissues because unable to repair damaged DNA quickly, while the biological complexity and heterogeneity of cancer result in inherent radioresistance (Mendenhall et al., 2001). The survival of HNSCC patients is restricted by existence of inherent radioresistant tumor cells and radioresistance after fractional radiotherapy, which leading to local recurrence and distant metastasis. When surgery is performed as a salvage method after radiotherapy failure, the rate of complications increase (Aarts et al., 2011). Therefore, radioresistance of HNSCC has become an important factor restricting the clinical efficacy of HNSCC and prolonging the survival of patients.

The mechanism of radioresistance in HNSCC has also become a recent research hotspot. The disruption of proliferation, apoptosis, DNA damage response and repair, angiogenesis, epithelial-mesenchymal transition and cancer stem cells are known as contributors to radioresistance in HNSCC (Chang et al., 2013; Brown, 2014; Barker et al., 2015; Ahmad et al., 2017). TP53 mutations were associated with apoptosis and increased radioresistance in HNSCC (Skinner et al., 2012). The most frequently mentioned pathways leading to the development of HNSCC radioresistance are EGFR, PI3K/AKT, and RAS Pathways (Zimmermann et al., 2006; Chang et al., 2013; Perri et al., 2015). Although there have been studies to explore the mechanism of radioresistance, the exact mechanism of radioresistance in HNSCC is still not completely clear up to now.

The clinical efficiency of HNSCC is greatly restricted by radioresistance. For exploring the characteristics and mechanism of radioresistant HNSCC, differentially expressed genes (DEGs) association with radioresistance were investigated in HNSCC using transcriptome analysis and public databases. The present study provides new markers for predicting radioresistance or targets for improving radiosensitivity.

## Materials and methods

### Cell culture and establishment of radioresistant HNSCC cells

Human laryngeal squamous carcinoma cell line, Hep2 was purchased from the Institute of Biochemistry and Cell Biology of the Chinese Academy of Sciences, Shanghai, China. The cells were cultured with RPMI medium 1,640(Corning, United States) containing 10% fetal bovine serum (Corning, United States) and 1% antibiotics (Gibco-BRL, Gaithersburg, MD, United States), and incubated at 37 °C with saturated humidity and 5% CO2. Radioresistant Hep2 (termed Hep2-R) was established by exposure repeated radiation of Hep2 with 8 Gy for 6 times (Zhou et al., 2020). For Hep2-R the D0 was 2.512 Gy, n was 2.52, Dq was 2.322 Gy, and D37 was 4.83 Gy, which compared to 1.879 Gy, 2.003,1.305 Gy, and 3.185 Gy for Hep2.

### Viability of radioresistant HNSCC cells

The viability and proliferation of above cells were verified by MTT assay, colony formation assay and flow cytometry. All experiments were carried out in five samples.

Cell proliferation was monitored by use of 2-(4,5-dimethyltriazol-2-yl)-2,5-diphenyl tetrazolium bromide (MTT, Sigma) assay. Briefly, Hep2 and Hep2-R were re-seeded into 96-well plate with 2 × 10^3^ cells per well and culture for 6 h. Exposed to 8 Gy and indicated for 72 h, 20 μL of MTT was added to each well and incubated at 37 °C with 5% CO2 for 4 h. After adding 150 μL of dimethyl sulfoxide, the optical density at 450 nm was measured for each sample using a Bio-rad 550 Microplate Reader.

In the colony formation assay, Hep2 and Hep2-R were re-seeded in 6-well plates at a density of 1 × 10^3^ cells per well and exposed to 8 Gy. The cells were cultured for 14 days then washed twice with PBS, fixed with methanol for 15 min, and stained with 0.1% crystal violet solution for another 20 min at room temperature. Five randomly chosen fields were taken by an inverted microscope to count the number of colonies, and the mean was taken.

An apoptosis assay was detected by AnnexinV-FITC/PI kit, and flow cytometry analysis was conducted using a BD LSR II flow cytometer. After exposing to 8 Gy and indicating for 72 h, Cells were collected and resuspended. The process was performed according to the manufacturer’s instructions.

### DEGs identification using microarray

According to the manufacturer’s protocol, total RNA was extracted from HNSCC radioresistant cell line Hep2-R and parental cell line Hep2 using TRIzol (Invitrogen, Carlsbad, CA, United States). Total RNA was tested integrity and construction by Agilent 2,100 Bioanalyzer (RIN≥7.0 and 28S/18S>0.7) and Thermo NanoDrop 2000 (1.7 < A260/A280 < 2.2). After constructing a RNA library for sequencing, cDNA was prepared by GeneChip WT PLUS Kit. The cDNA was purified and segmented, and hybridized with the chip probe. The chip was washed and dyed, and images and original data were obtained by scanning After the hybridization.

### Identifying DEGs using databases

In Gene Expression Omnibus (GEO) database (https://www.ncbi.nlm.nih.gov/geo), GSE9712, GSE9713 and GSE9714 are based on the platform of GPL 96, detecting DEGs in radioresistant and radiosensitive tumors and cell lines in HNSCC. GSE9712, GSE9713 and GSE9714 belong to SuperSeries GSE9716. GSE9712 detected DEGs in radioresistant tumors, GSE9713 Detected DEGs in radioresistant and radiosensitive tumors before and after irradiation, and GSE9714 explored DEGs in radioresistant and radiosensitive human head and neck tumor cell lines. Radiosensitive human head and neck tumor cell lines is SCC-61. Nu61, a radioresistant human head and neck tumor cell lines, was selected from a parental radiosensitive tumor SCC-61 by eight serial cycles of passage in athymic nude mice and *in vivo* irradiation. Obtained tumors were established as xenografts in nude mice using SCC-61 and Nu61 (Khodarev et al., 2004; Khodarev et al., 2007). GSE67614 was used to validate the findings, containing 102 HNSCC specimens from surgically excised tumors. These specimens are from patients treated by post-operative radiation therapy and are being used to develop gene signatures for therapeutic response. For identifying DEGs, both R and GEO2R were used (https://www.ncbi.nlm.nih.gov/geo/geo2r/). Genes with |log FC| ≥ 1 and *p* < 0.05 were regarded as DEGs.

### The prognostic value and protein expression of FN1

The prognostic value of FN1 was obtained from the gene expression profiling interactive analysis (GEPIA) database (http://gepia.cancer-pku.cn/index.html). The protein location of FN1 was detected using human protein atlas (HPA).

### Statistical analysis

All statistical analyses were analyzed using a SPSS (version 24.0; SPSS Inc., Chicago,IL). Data are presented as the mean ± standard deviation. Student’s t-test was used to compare the differences between the two groups. Statistical significance was set at *p* < 0.05.

## Results

### Hep2-R was successfully established by intermittent and repeated sub-lethal dose X-ray radiation

**FIGURE 1:**
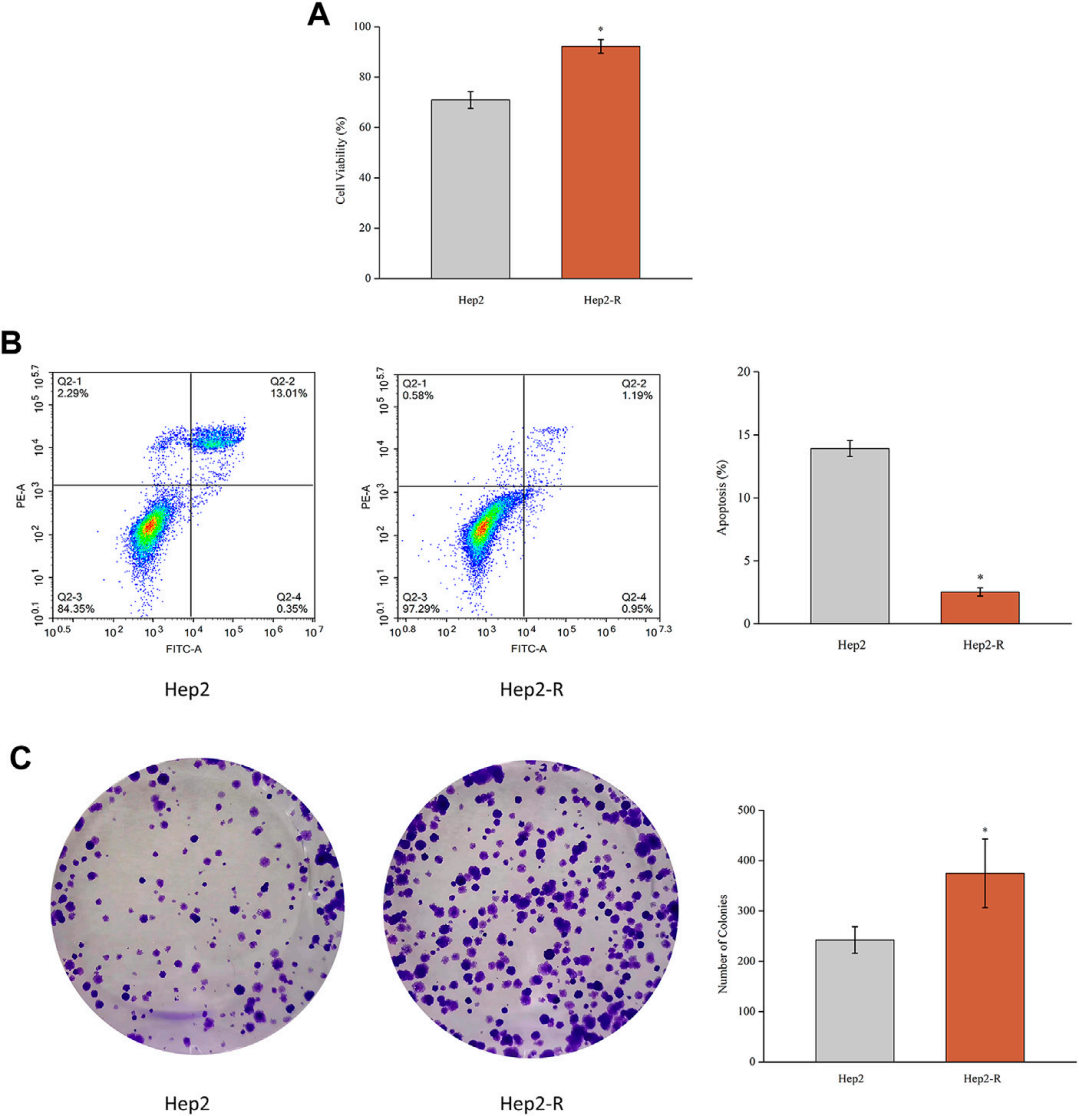
**(A)** MTT assay was performed to measure the viability of Hep2 and Hep2-R. **(B)** Apoptosis of Hep2 and Hep2-R were detected on flow cytometry. **(C)** The clone formation rate of Hep2-R was higher than Hep2. *p < 0.05.

To explore cell proliferation and radioresistant capacity after exposure to radiation, MTT assay and colony formation assay were applied. As shown in Figure 1A, the proliferation rate of Hep2-R was higher than that of the control group (*p* < 0.05). As shown in Figure 1C, after explosion under the same conditions, the clone formation rate of Hep2-R was higher than that of the control group (*p* < 0.05). Furthermore, Hep2 showed significant apoptosis compared to Hep2-R (Figure 1B). These results indicated that Hep2-R decreased the sensitivity of Hep2 to X-ray irradiation.

### Radioresistance causes a differential gene expression profile

**FIGURE 2:**
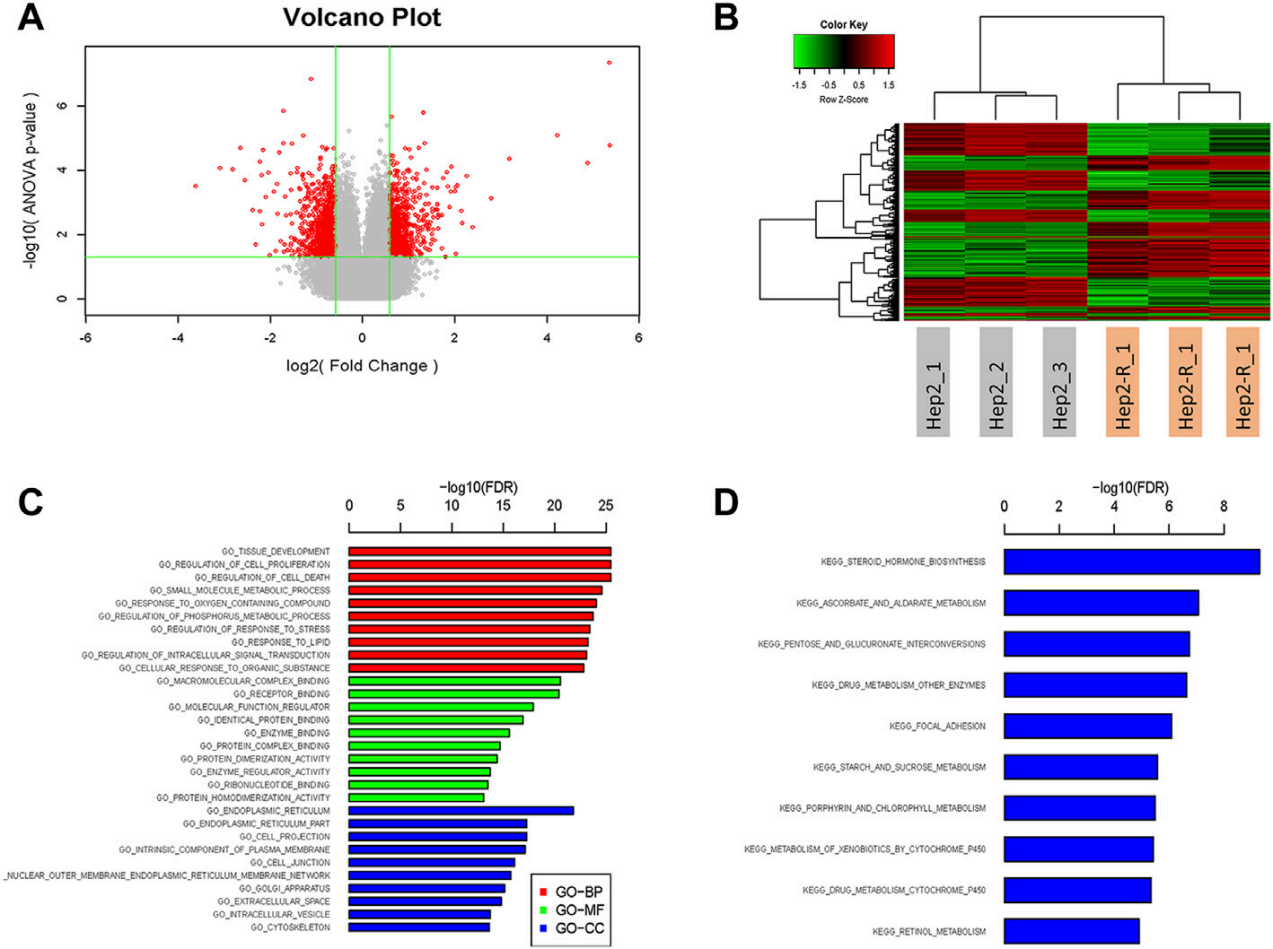
Volcano plot **(A)**, Hierarchical clustering **(B)**, GO analysis **(C)** and KEGG pathway analysis **(D)** of 2,360 differentially expressed genes.

To identify radioresistant differentially expressed genes (DEGs), RNA sequencing (RNA-seq) technology was used. 2,360 DEGs were identified (absolute average fold change >1.5 and *p* < 0.05), of which 1,144 were upregulated and 1,216 were downregulated. The names of these 2,360 DEGs and their average fold change are shown in Supplementary Table S1 and volcano plot was showed in Figure 2A.

### Hierarchical clustering, gene-ontology and kyoto encyclopedia of genes and genomes pathways analysis of the differential expressed genes

To get more insight on the biological significance of the radioresistant DEGs, hierarchical clustering was performed. All DEGs were hierarchically grouped into four groups (Figure 2B). The genes within the same cluster are coregulated genes, and may have similar biological functions during bronchial epithelial carcinogenesis. GO analysis revealed that DEGs are enriched with the genes of different functions, such as tissue development, regulation of cell proliferation (Figure 2C). KEGG pathway analysis showed that DEGs involved in 10 signaling pathways (Figure 2D). The DEGs may play a role in radioresistance by these signaling pathways. The DEGs were enriched in pathways including “steroid hormone biosynthesi”, “ascorbate and aldarate metabolism”, “pentose and glucuronate interconversions”, “drug metabolism—other enzymes”, “focal adhesion”, “starch and sucrose metabolism”, “porphyrin and chlorophyll metabolism”, “metabolism of xenobiotics by cytochrome P450”, “drug metabolism—cytochrome P450”, and “retinol metabolism”.

### Identifying biomarkers in radioresistant HNSCC cells and tissues

**FIGURE 3:**
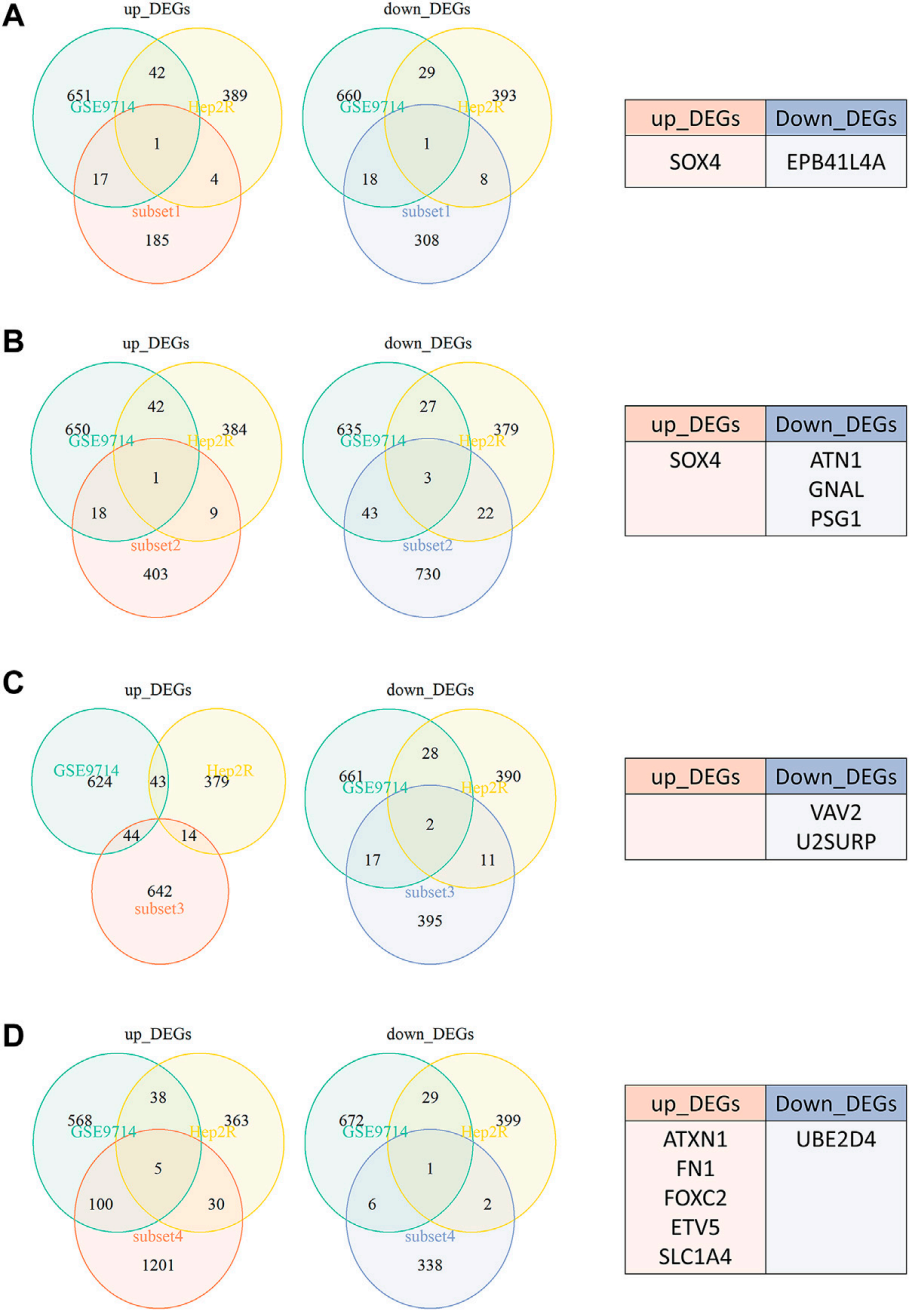
The Venn diagram revealed that a total of 13 DEGs were found when our data (Hep2 versus Hep2-R) and GSE9714 compared with four subsets. **(A)** Subset 1-six radiosensitive tumors versus six radioresistant tumors without irradiation. **(B)** Subset 2-three radiosensitive tumors versus three radioresistant tumosr with irradiation. **(C)** Subset 3-three radiosensitive tumors versus three radioresistant tumors 5 h after irradiation. **(D)** Subset 4-three radiosensitive tumors versus three radioresistant tumors 24 h after irradiation.

We previously constructed HNSCC radioresistant cell line, Hep2-R, by repeated X-ray irradiation on Hep2. In addition, we also analyzed the DEGs between radioresistant HNSCC cells and tissues using GEO dataset SuperSeries GSE9716, including GSE9712, GSE9713 and GSE9714. In GSE9714, two radioresistant and two radiosensitive HNSCC cell lines were selected. All tumor samples between GSE9712 and GSE9713 were divided into four subsets according to irradiation: Subset 1 (six radiosensitive tumors versus six radioresistant tumors without irradiation, Figure 3A), Subset 2 (three radiosensitive tumors versus three radioresistant tumosr with irradiation, Figure 3B), Subset 3 (three radiosensitive tumors versus three radioresistant tumors 5 h after irradiation, Figure 3C), Subset 4 (three radiosensitive tumors versus three radioresistant tumors 24 h after irradiation, Figure 3D). The Venn diagram revealed that a total of 13 DEGs were found when our data (Hep2 versus Hep2-R) and GSE9714 compared with above four subsets. Six genes were overexpressed and seven genes were lowly expressed in radioresistant cells and tissues in comparison with the normal with absolute average fold change >1 and *p* < 0.05 (Figure 3).

### Upregulation of FN1, SOX4 and ETV5 contributes to the radioresistance of HNSCC

**FIGURE 4:**
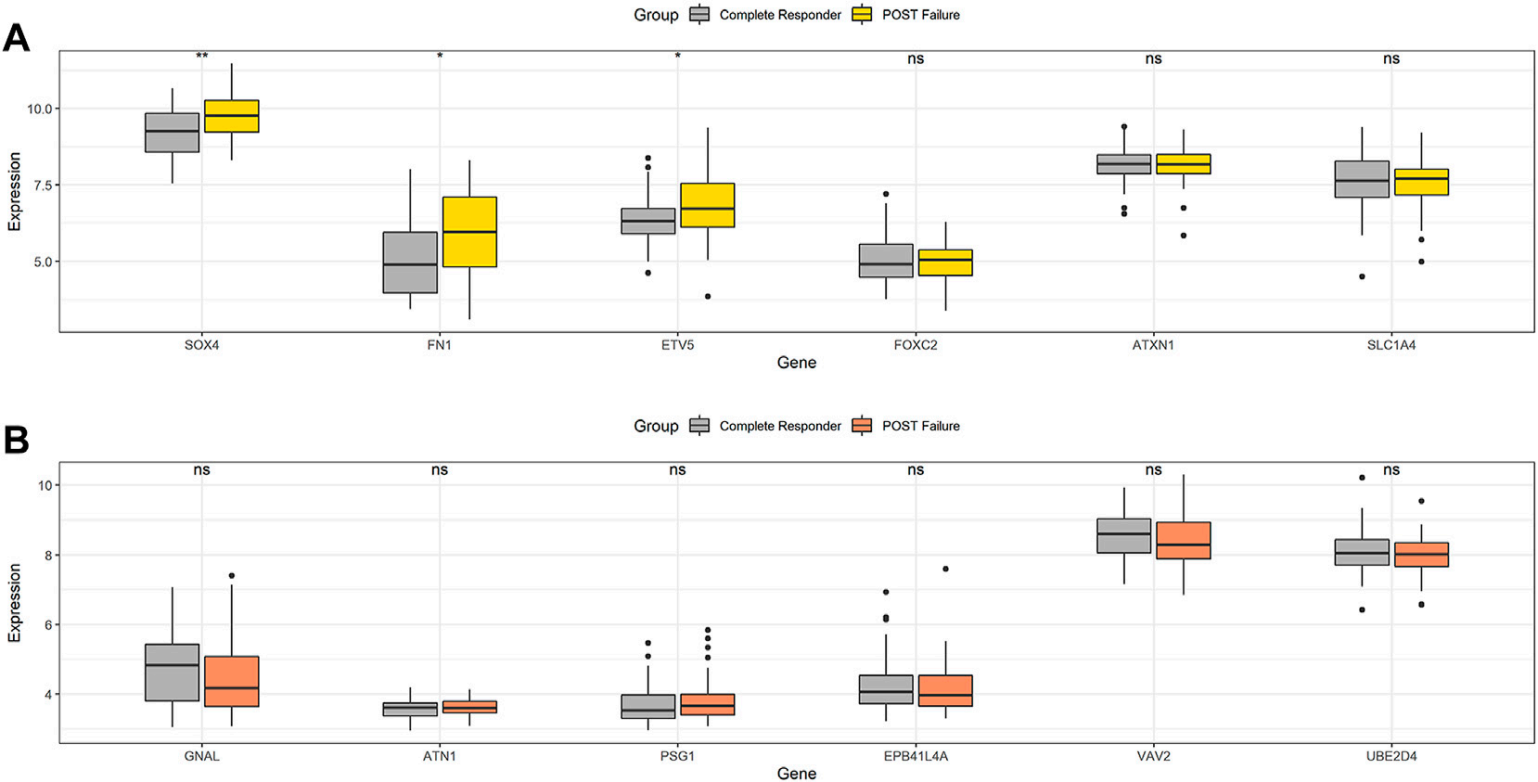
In GSE67614, tumor tissues with post-operative radiation therapy failure had higher expression of upregulated radioresistant genes FN1, SOX4 and ETV5 **(A)**, while none of the downregulated radioresistant genes were downregulated simultaneously **(B)**. *p < 0.05, **p < 0.01.

To verify this deduction, we also analyzed the 13 DEGs in surgically excised tumor tissues between radiosensitive and radioresistant patients in GEO dataset in GSE67614. Except U2SURP, 12 DEGs were identified in GSE67614.50 tumor tissues from HNSCC radiosensitive patients and 52 from radioresistant HNSCC patients were selected. All HNSCC patients had surgery and post-operative radiation therapy. Figure 4A showed that tumor tissues with post-operative radiation therapy failure had higher expression of upregulated radioresistant genes FN1, SOX4 and ETV5, while none of the downregulated radioresistant genes were downregulated simultaneously (Figure 4B). Therefore, we speculated that high expression of FN1, SOX4 and ETV5 might be important biomarkers of HNSCC radioresistance. The above three proteins affected the radiosensitivity of HNSCC *in vivo*.

### High level of FN1 is a prognostic indicator of HNSCC

**FIGURE 5:**
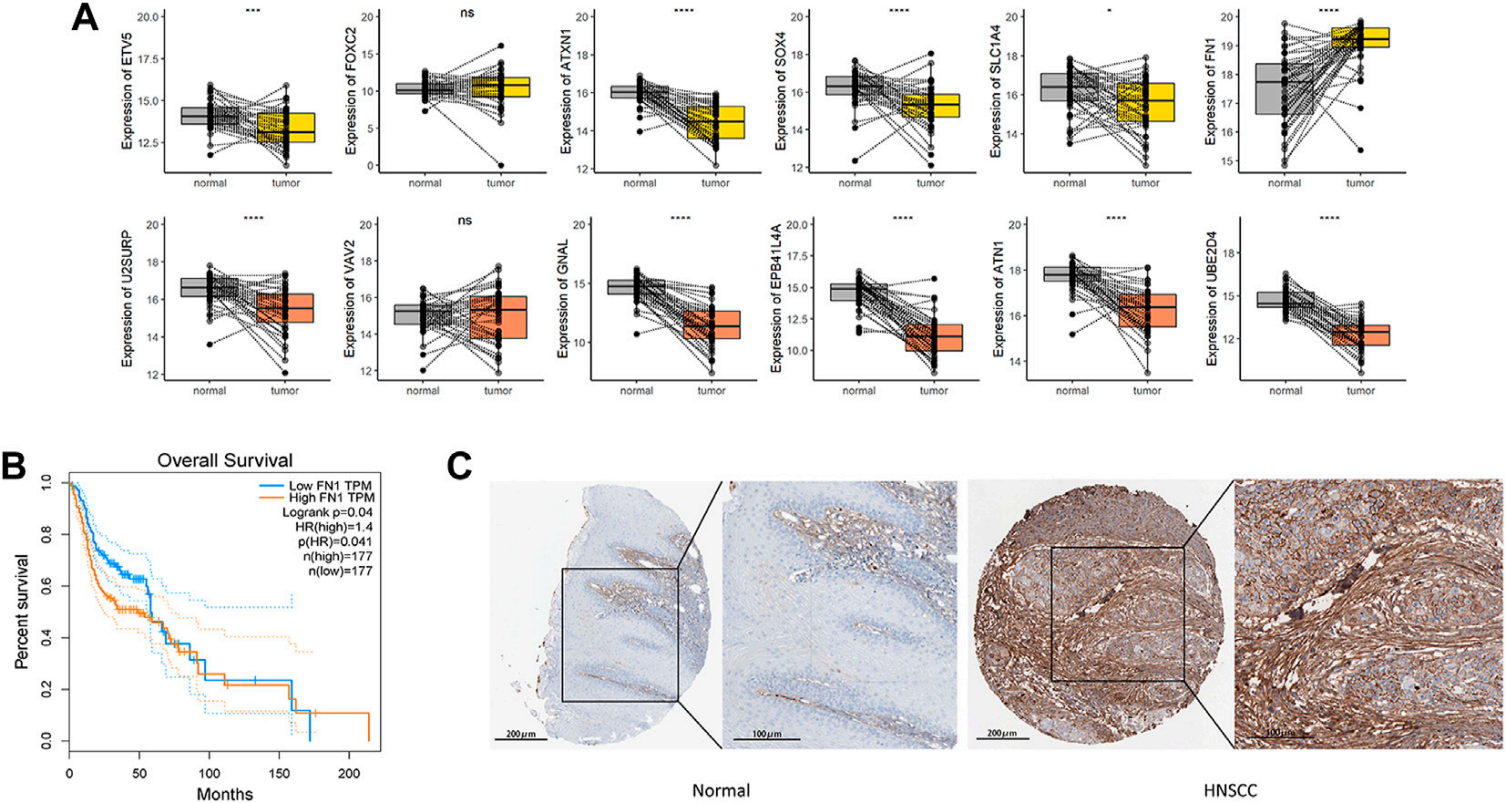
**(A)** In HNSCC TCGA dataset, overexpression of FN1, SOX4 and ETV5 were found in 43 paired samples between paracancerous tissues and tumor tissues. **(B)** While only FN1 was associated with prognosis when all HNSCC samples in TCGA dataset were included. **(C)** FN1 overexpressed in HNSCC tissues, meaning protein expression was consistent with mRNA expression. ***p < 0.001, ****p < 0.0001.

**FIGURE 6:**
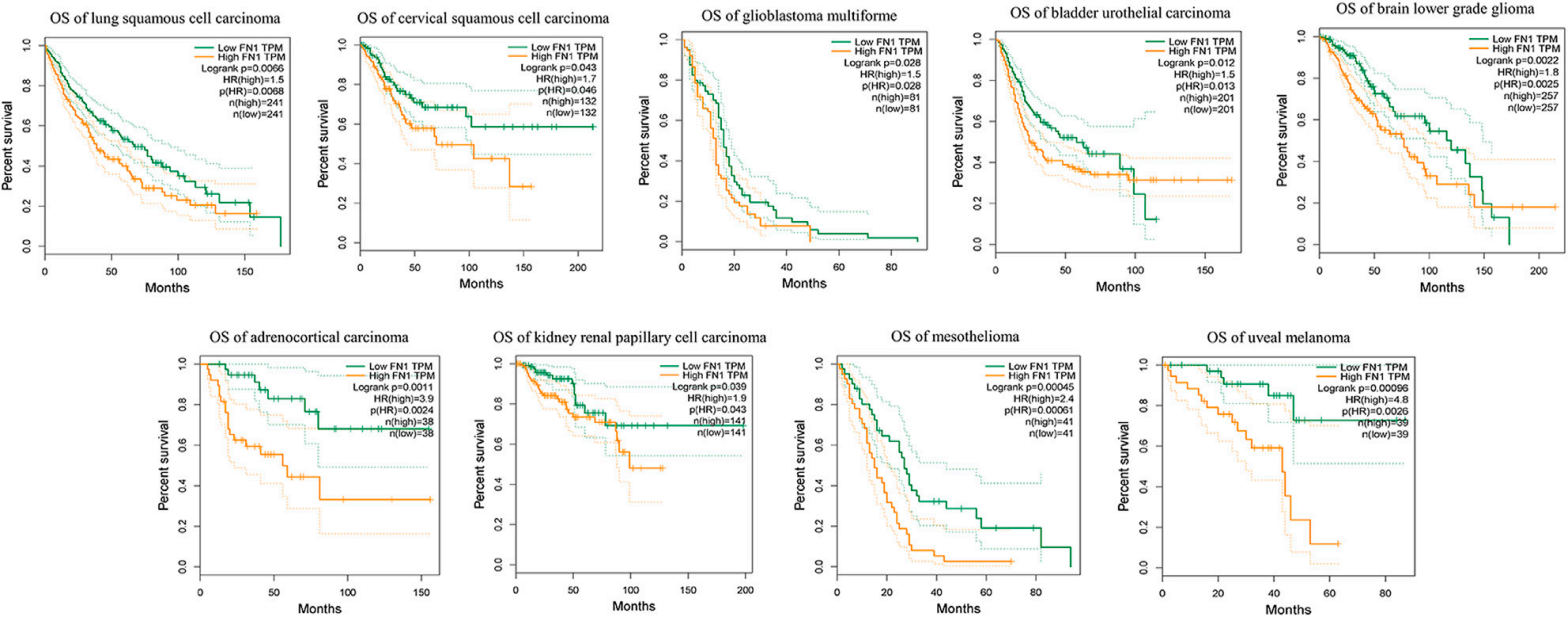
As a risk factor, FN1 not only affected the prognosis of patients with HNSCC, but also affects other types of squamous cell carcinomas or other types of carcinomas in TCGA dataset.

Aiming to understand the association of above DEGs (FN1, SOX4 and ETV5) with HNSCC prognosis, the alterations of them were explored through HNSCC TCGA dataset. Overexpression of FN1, SOX4 and ETV5 were found in 43 paired samples between paracancerous tissues and tumor tissues of HNSCC (Figure 5A). While only FN1 was associated with prognosis when all HNSCC samples in TCGA dataset were included (Figure 5B). As a risk factor, FN1 not only affected the prognosis of patients with HNSCC, but also affects other types of squamous cell carcinomas (lung squamous cell carcinoma and cervical squamous cell carcinoma) or other types of carcinomas in TCGA dataset (Figure 6). Figure 5C showed that FN1 overexpressed in HNSCC, meaning protein expression was consistent with mRNA expression in HNSCC tissues.

## Discussion

Radioresistance restricts the prognosis of HNSCC, therefore it is crucial to find biomarkers and therapeutic targets associated with radiosensitivity. In the present study, differential expression profile of genes associated with radioresistance in HNSCC was established using transcriptome analysis. Based on DEG analysis of the HNSCC radioresistant cells and tissues in public databases, 13 DEGs were identified. FN1, SOX4 and ETV5 were further validated in HNSCC patients’ tumor tissues with irradiated failures, which is novel radioresistance biomarkers in HNSCC. Furthermore, a comprehensive database analysis showed that FN1 had a high expression level between HNSCC patients with post-operative radiation therapy failure and tumor tissues of HNSCC from the GEO and TCGA. From GEPIA, patients harboring an increased expression of FN1 not only have a poor prognosis in HNSCC, but also in other 2 types of squamous cell carcinomas, such as lung squamous cell carcinoma and cervical squamous cell carcinoma. Considering the level of FN1 under different conditions, it is rational that FN1 affects the prognosis of HNSCC, and radiation could enhance the transcriptional level of FN1.

KEGG, as an integrated database, provides networks of generic molecular and understanding of disease mechanisms (Kanehisa et al., 2021). The mechanisms underlying HNSCC radioresistance not yet been fully elucidated. In our study, mechanism was analyzed with KEGG. The DEGs were enriched in pathways including “steroid hormone biosynthesi”, “ascorbate and aldarate metabolism”, “pentose and glucuronate interconversions”, “drug metabolism—other enzymes”, “focal adhesion”, “starch and sucrose metabolism”, “porphyrin and chlorophyll metabolism”, “metabolism of xenobiotics by cytochrome P450”, “drug metabolism—cytochrome P450”, and “retinol metabolism”. These results suggested that cell adhesion, cytochrome P450 and drug metabolism were associated with HNSCC radioresistance. Focal adhesion signaling includes integrins and epidermal growth factor receptors (EGFR), several kinases and adapter molecules, which impacted on contact and connection between cell and extracellular matrix (ECM) and contributed to tumor development and progression (Eke and Cordes, 2015). Integrins and EGFR not only regulate tumorigenesis and metastasis but also modulate radioresistance. Recent studies illustrate β1 integrin inhibition enhanced radiosensitivity in HNSCC, mainly impacting on β1 Integrin/FAK/cortactin signaling (Eke et al., 2012a; Eke et al., 2012b). αV integrin enhances radioresistance in human nasopharyngeal carcinoma *via* SAPK/JNK pathway (Ou et al., 2012). Overexpression of EGFR indicates poor prognosis, and targeted agents of EGFR enhanced radiosensitivity (Bonner et al., 2006; Cohen, 2014). The Molecular interplays between integrin and EGFR cannot be ignored. EGFR and integrin in astrocytoma frozen sections predict clinical outcome and correlate with radioresistance *in vitro* (Petras et al., 2013). The efficacy of EGFR inhibition in HNSCC cells can be enhanced by joint targeting of EGFR and β1 integrin (Eke and Cordes, 2011; Eke et al., 2013).

Our study revealed 19 DEGs enriched in focal adhesion, including Fibronectin 1 (FN1). FN1 locates in both cell surface and extracellular matrix of multiple cell types, linking matrices and cells. As a member of the fibronectin family, FN1 involves in embryologic development, wound healing, hematopoiesis (Wang and Ni, 2016) and infections (Speziale et al., 2019). FN1 in the tumor cells is called cancerous fibronectin, and related to malignancy, metastasis or poor prognosis of tumor (Steffens et al., 2012; Lin et al., 2020; Sun et al., 2020). FN1 interacted with binding partner ITGA5 and promoted viability, invasion, and migration in colorectal cancer through suppressing apoptosis (Sun et al., 2020). FN1 was bound by miR-1271 in neuroglioma (Gong et al., 2017). In HNSCC, several studies have proved FN1 was potential diagnostic indicators and involved in focal adhesion signaling pathway (Li et al., 2019) using integrated bioinformatics methods (Kuang et al., 2016). However, the importance of FN1 on the HNSCC radiation resistance is still unclear. Our study identified FN1 was an up-regulated genes related to worse prognosis and radioresistance, and enriched in focal adhesion pathway. These results suggested that FN1 overexpression is an indicator for poor prognosis and radioresistance, and focal adhesion may be associated with radioresistance in HNSCC.

In this study, SOX4 and ETV5 showed association with radioresistance in HNSCC. SOX4 belongs to the C subgroup of the SOX family, which involve in many developmental processes. High-expressed SOX4 affected tumor development (Fang et al., 2012; Zhang et al., 2012; Wang et al., 2013) or radioresistance (Xue et al., 2021) in many cancers. In HNSCC, Study from Yoon et al. (Yoon et al., 2015) proved that SOX4 may serve as an oncogene and causes radioresistance. E26 transformation-specific (ETS) transcription factors including ETV5, play important roles in tumor cell invasion, differentiation and angiogenesis (de Sousa et al., 2014). It has been reported that high levels of ETV5 facilitate tumor cell proliferation, epithelial-mesenchymal transition, and metastasis in ovarian and colorectal cancers (Llauradó et al., 2012; Cheng et al., 2019). The relation between ETV5 and radioresistant HNSCC is unclear, but ETV5 can overcome cetuximab resistance. Park et al. found that knockdown of ETV5 can increase cetuximab sensitivity in colon cancer cell lines (Park et al., 2019).

The important aspect of this study proved that overexpressed FN1 is correlated with radioresistance in HNSCC and enriched in focal adhesion pathway. Nevertheless, our study has limitations. The mechanisms underlying HNSCC radioresistance not yet been fully elucidated, additional studies *in vivo* and *in vitro* are needed to verify the actual value of the FN1. The present study provides important insights into novel prognostic indicators for patients with HNSCC radioresistance.

## Conclusion

In summary, we established an expression profile of genes associated with radioresistance in HNSCC cells and tissues, and found that FN1 was overexpressed in the HNSCC patients with radiation failure, suggesting that FN1 may contribute to tumorigenesis and radioresistance the in HNSCC. FN1 most likely involved in focal adhesion to regulate radiosensitivity. FN1 is an indicator for prognostic and radioresistamce in HNSCC patients.

## Data Availability

The datasets presented in this study can be found in online repositories. The names of the repository/repositories and accession number(s) can be found in the article/Supplementary Material.
